# Primary care *I*dentification and *R*eferral to *I*mprove *S*afety of women experiencing domestic violence (IRIS): protocol for a pragmatic cluster randomised controlled trial

**DOI:** 10.1186/1471-2458-10-54

**Published:** 2010-02-02

**Authors:** Alison Gregory, Jean Ramsay, Roxane Agnew-Davies, Kathleen Baird, Angela Devine, Danielle Dunne, Sandra Eldridge, Annie Howell, Medina Johnson, Clare Rutterford, Debbie Sharp, Gene Feder

**Affiliations:** 1 Academic Unit of Primary Health Care, University of Bristol, 25-27 Belgrave Road, Clifton, Bristol, BS8 2AA, UK; 2 Barts and The London School of Medicine and Dentistry, Queen Mary University of London, Centre for Health Sciences, Abernethy Building, 2 Newark Street, Whitechapel, London, E1 2AT, UK; 3 Domestic Violence Training Ltd, 12 Hook Road, Surbiton, Surrey, KT6 5BH, UK; 4 School of Health and Social Care, University of the West of England, Frenchay Campus, Coldharbour Lane, Bristol, BS16 1QY, UK; 5 The Nia Project, Unit 2J Leroy House, 436 Essex Road, London, N1 3QP, UK; 6 Next Link, 5 Queen Square, Bristol, BS1 4JQ, UK

## Abstract

**Background:**

Domestic violence, which may be psychological, physical, sexual, financial or emotional, is a major public health problem due to the long-term health consequences for women who have experienced it and for their children who witness it. In populations of women attending general practice, the prevalence of physical or sexual abuse in the past year from a partner or ex-partner ranges from 6 to 23%, and lifetime prevalence from 21 to 55%. Domestic violence is particularly important in general practice because women have many contacts with primary care clinicians and because women experiencing abuse identify doctors and nurses as professionals from whom they would like to get support. Yet health professionals rarely ask about domestic violence and have little or no training in how to respond to disclosure of abuse.

**Methods/Design:**

This protocol describes IRIS, a pragmatic cluster randomised controlled trial with the general practice as unit of randomisation. Our trial tests the effectiveness and cost-effectiveness of a training and support programme targeted at general practice teams. The primary outcome is referral of women to specialist domestic violence agencies. Forty-eight practices in two UK cities (Bristol and London) are randomly allocated, using minimisation, into intervention and control groups. The intervention, based on an adult learning model in an educational outreach framework, has been designed to address barriers to asking women about domestic violence and to encourage appropriate responses to disclosure and referral to specialist domestic violence agencies. Multidisciplinary training sessions are held with clinicians and administrative staff in each of the intervention practices, with periodic feedback of identification and referral data to practice teams. Intervention practices have a prompt to ask about abuse integrated in the electronic medical record system. Other components of the intervention include an IRIS champion in each practice and a direct referral pathway to a named domestic violence advocate.

**Discussion:**

This is the first European randomised controlled trial of an intervention to improve the health care response to domestic violence. The findings will have the potential to inform training and service provision.

**Trial registration:**

ISRCTN74012786

## Background

### The scale of the problem

Domestic violence is threatening behaviour, violence or abuse (psychological, physical, sexual, financial or emotional) between adults who are in the same family or who are (or have been) intimate partners. The prevalence of physical and sexual violence varies internationally from 15 to 71% [1]. The 2001 British Crime Survey reported that 20% of women in England and Wales were physically assaulted by a current or former partner at some time in their lives. When threats, financial abuse and emotional abuse are included, this increased to 25% of women [2]. The prevalence of domestic violence among women seeking healthcare is higher than the general population. In populations of women attending general practice, the prevalence of physical or sexual abuse in the past year from a partner or ex-partner ranges from 6 to 23%, and lifetime prevalence from 21 to 55% [3]. The two fold variation reflects different measures or abuse, as well as national and regional differences. In a questionnaire survey of 1035 women attending east London general practices the prevalence of physical abuse in the past year from a partner or ex-partner ranges was 17% and lifetime prevalence 40% [4]. The 2008 costs of domestic violence to the UK economy, including services, loss of economic output, and human and emotional costs is £16 billion annually [5].

### Health consequences

Domestic violence damages health. Survivors suffer many chronic health problems including: gynaecological problems [6], chronic pain and neurological symptoms [7], gastrointestinal disorders [8,9], and self-reported cardiovascular conditions [10]. Domestic violence may start or escalate during pregnancy [11,12] with the most serious outcomes being the death of the mother [13] or the foetus [14,15] and a consistent association with low birth weight, taking into account parity, age and socioeconomic status [16,17]. There is overwhelming evidence for the impact of domestic violence on mental health [2], which can last long after the violence has ceased. The most prevalent mental health sequelae of domestic violence are depression and post-traumatic stress disorder (PTSD), with parallels to the trauma of being taken hostage and subjected to torture [16,18-23]. Coid and colleagues' study of women with a lifetime experience of domestic violence attending general practices in London, found odds ratios greater than three for depression, anxiety and PTSD, and greater than two for suicide attempt, use of illegal drugs and alcohol abuse [24]. Women experiencing domestic violence present frequently to health services and require wide-ranging medical care [25,26]. Witnessing of domestic violence by children results in developmental problems and long term mental health, educational and social sequelae [27-29].

### Current health care response is inadequate

Healthcare may be a survivor's first or only point of contact with professionals [30] and abused women are more likely to be in touch with health services than any other agency [31]. Eighty percent of women in a violent relationship seek help from health services at least once [32] and women suffering from the effects of domestic violence typically make 7-8 visits to health professionals, either on their own or on someone else's behalf, before disclosure of abuse [33].

The magnitude of the health consequences of domestic violence contrasts starkly with its virtual invisibility within primary care; in one general practice based questionnaire study only 15% of women with a history of domestic violence had any reference to violence in their medical record [34]. Specialist domestic violence agencies receive few referrals from health care services. If women disclose domestic violence to a clinician, whether in a primary care or specialist setting, there is evidence of an inappropriate, poor quality response [35]. Doctors and nurses in general are largely unaware of appropriate interventions and have seldom received effective or, indeed, any training [36]. Yet the issue of domestic violence is particularly important in this context because of the extensive contact between women and primary care clinicians (90% consult in 5 years), and because abused women themselves identify primary care clinicians as the people from whom they would seek support [37].

### Evidence for effectiveness of system level interventions and specialist domestic violence advocacy

There is evidence that system level training and organisational change can increase the disclosure of domestic violence to healthcare professionals, although this conclusion is largely based on non-randomised North American studies [38] and is not consistently found in randomised trials [39]. There is even less evidence that clinician training or organisational change results in better care or referral to specialist domestic violence services [38], and the only trial to date measuring women centred outcomes (reoccurrence of violence or quality of life) of a system level screening intervention found no significant differences between intervention and control arms [40].

Referral to domestic violence services that provide specialist advocacy is a proxy outcome for interventions aiming to improve the health service response to domestic violence. In the context of domestic violence services, advocacy is a term that varies within and between countries, depending on institutional settings and historical developments of the role of advocates. Advocates engage with individual clients who are being abused, aiming to empower them and linking them to community services. In some health settings they may also have a role in bringing about system change, catalysing increased recognition by clinicians of women experiencing abuse. Core activities of advocacy include: provision of legal, housing and financial advice, facilitating access to and use of community resources, such as refuges (shelters, safe houses), emergency housing, and provision of safety planning advice. Advocates can also provide ongoing support and informal counseling. In a 2009 Cochrane review confined to randomized controlled trials of domestic violence advocacy, Ramsay and colleagues concluded that there was equivocal evidence that advocacy for women recruited in domestic violence shelters (refuges) had a beneficial effect on their physical and psychosocial well-being, and were unable to draw any conclusions for women receiving advocacy in or referred from health care settings [41]. In a broader systematic review which included all controlled studies of domestic violence advocacy, Feder and colleagues reported that most showed improvement in some women-centred outcomes. In particular, for women who have actively sought help from professional services, advocacy can reduce abuse, increase social support and quality of life, and lead to increased usage of safety behaviours and accessing of community resources [42]. Five of the eleven studies had participants recruited within health care settings [43-49].

In summary, domestic violence is a major public health problem with a poor response from health care services. There is insufficient evidence for system level interventions to improve this response and there is a need to rigorously test such interventions.

This paper describes the protocol of a cluster randomised controlled trial of a domestic violence training and support programme targeted at general practice teams, comparing the outcomes for practices that receive the intervention to those that do not.

## Methods/Design

### Primary Objective

• To determine the effectiveness of an intervention delivered in general practice, designed to improve recording and management of domestic violence in primary care, compared with usual practice in terms of the rate of referral to a specialist domestic violence agency providing advocacy.

### Secondary Objectives

• To determine the effectiveness of an intervention delivered at the general practice level, designed to improve recording and management of domestic violence in primary care, compared to usual practice in terms of the rate of domestic violence disclosure in relation to the number of women aged 16 or older registered in the practice.

• To estimate the cost effectiveness of the intervention.

• To determine whether individual physician attitudes, knowledge and behaviour in relation to domestic violence change after the intervention.

### Practice recruitment

General practices in two primary care trusts in the south west of England (Bristol) and east London (Hackney) are eligible for participation if they have EMIS LV, EMIS PCS or iSoft Synergy electronic medical record systems. These systems allow the use of clinician prompts and templates within the medical record and were the dominant systems in both areas (>90%).

We have obtained lists of practices in two primary care trusts in the south west of England (Bristol) and east London (City and Hackney) and stratified them by practice characteristics: number of whole time equivalent doctors, training status and presence of a practice counselor. All practices on the lists with the relevant record systems were considered eligible for the trial except for two in which investigators were practicing. A stratified random sample of the general practices in each site was generated from random number tables and those practices were then contacted in rank order. General practices received a postal invitation to participate, including details of the study and a copy of an editorial from a general practice journal highlighting domestic violence as a central primary care issue [50]. The invitation was followed up within a fortnight by telephone contact with one of the general practitioners (GF in London and DS in Bristol) to discuss possible participation. Practices where initial interest in the study was expressed were posted or e-mailed a further information sheet, and given time to consider participation in the trial. Practices wishing to have further details or clarification were offered a practice visit to explain the study in more detail. Implicit consent from individual clinicians within recruited practices was assumed from their subsequent attendance at training sessions and their completion of study questionnaires.

### Study design and practice allocation

As the intervention was targeted at the practice teams (clinicians and reception staff), the trial is cluster randomised with the practice as unit of analysis. The 48 practices recruited in both Hackney and Bristol were allocated (by JR for Hackney practices and DD for Bristol practices) to intervention and control arms of the trial with a computer minimisation programme, including a random component (Minim Version 1.3). Allocation was determined by the programme and the process was concealed from the person entering practice minimisation variables. The variables were the percentage of female doctors in the practice (whole time equivalents), postgraduate training status, the number of patients registered with the practice, and the percentage of the practice population on low incomes.

### Intervention

The training and support intervention within an educational outreach framework [51], has a theoretical basis in adult learning theory and peer influence [52]. It focuses on addressing the barriers to asking women patients about abuse and responding appropriately, which includes offering referral to a specialist domestic violence agency that provided advocacy. The primary intervention consisted of two 2-hour multidisciplinary training sessions, scheduled at lunchtime on practice premises, targeted at the clinical team: general practitioners, directly employed practice staff (practice nurses and counselors), and those employed by primary care trusts (midwives, health visitors, district nurses) who have contact with patients registered in the practice. The training sessions were designed to address the expressed and tacit barriers to improving the response of clinicians to women experiencing abuse through improved identification, support and referral to specialist agencies. These sessions incorporated case studies and role play in relation to asking about violence and responding appropriately. They were delivered by an advocate educator based in one of the two collaborating specialist agencies: Nia project in London http://www.niaproject.info/ and Next Link in Bristol http://www.nextlinkhousing.co.uk/, a clinical psychologist specializing in domestic violence and an academic general practitioner. The advocate educator was central to the intervention, combining a training and support role to the practices with provision of advocacy to women referred from the practices. The training sessions were followed by periodic contact with the practice in clinical meetings, feeding back anonymised practice data on disclosure and referral to the advocacy service, and reinforcing guidance on good practice with regards to domestic violence, as well as *ad hoc *telephone conversations with clinicians about referrals or advice. One hour training sessions with administrative staff focused on issues of confidentiality and safety for patients experiencing abuse and introduced the IRIS information materials signposting domestic violence agencies. Ongoing support to clinicians and reception staff in the practices was provided by the named domestic violence advocate educator, with the aim of consolidating the initial training

Intervention practices also were asked to identify a "champion" for the project; with the agreement of the practice, a member of staff from any of the clinical disciplines was invited to attend an additional two days training about domestic violence and to integrate this into the work of the practice.

Other components of the intervention include: a pop-up template linked to diagnoses (such as depression, anxiety, irritable bowel syndrome, pelvic pain and assault) which acts as a prompt to remind clinicians to ask questions about domestic violence and to record this in the electronic medical record, an explicit referral pathway to the named advocate in the Nia project and Next Link and publicity materials about domestic violence visible in the practices (waiting rooms and women's toilets). The template in the electronic medical record is based on HARK, four questions which have been validated for the identification of intimate partner violence in UK general practice [51].

Control practices are offered the training sessions once the follow-up period for the intervenrm of the trial is complete.

### Primary Outcome

Our primary outcome is the rate of referral to advocacy and/or specialist domestic violence agencies for one year following the educational sessions in intervention practices (measured for each practice in the intervention and control arms). A woman aged 16 or over is counted as a referral within the practice if her general practitioner records referral information. The denominator is the number of women aged 16 or over within the practice.

### Secondary Outcomes

Domestic violence disclosure rate for one year following the educational sessions in intervention practices: this is measured for each practice (intervention and control) from searches of the practice database for domestic violence codes in the electronic medical record. The denominator is the number of women aged 16 or above within each practice.

Resource use and associated costs of the intervention are collected for estimation of the IRIS programme's cost-effectiveness.

Physician Readiness to Manage Partner Violence Survey (PREMIS) [53]: this is completed by health professionals within each intervention and control practice at two time points (baseline and twelve months post intervention). We compare sum scores for perceived preparation, perceived knowledge, actual knowledge, opinions and self-reported practices.

### Data collection

Data are collected by research associates from the electronic medical records in each intervention practice twelve months after the second training session. This comprises identification and referral to domestic violence advocacy of women who have experienced or are experiencing domestic violence. We are searching over two time periods: (i) the twelve month period preceding the intervention, and (ii) the twelve month period after the second training session. Use of the trial template and/or domestic violence codes is identified by a series of computer searches, followed by examination of any eligible patient's electronic record that contains these codes, to determine identification or referral to any agency providing domestic violence advocacy. The same data are collected from the control group practices (the only exception being that identification data based on the use of the trial template codes will not be available). At the time of follow-up data collection for each intervention practice, data are collected from a control practice recruited at least 12 months prior to the data collection time point with similar practice size and training practice status to ensure comparability in the timing of data collection from similar practices.

Whether a record of identification or referral is present in a record or not, is determined using a detailed flowchart (available from authors) to reduce subjectivity. If the researchers extracting data from the medical record are uncertain about whether an identification or a referral was made or not, this will be referred to an independent outcomes panel, blinded to the arm of the trial and identity of the practice the case came from, for a decision. The panel members consist of two general practitioners (one with extensive domestic violence research experience) and a trial methodologist. Direct extracts from the medical record are presented to the table and decisions are made by majority voting if there is no unanimity. Validity of referral data collection will be assessed by an independent researcher searching and extracting from the records of four randomly selected practices in each of the two sites.

Some women may self-refer to various agencies for support or be referred by other professionals. As these referrals may be affected by our intervention (either negatively or positively), other sources of referral data will also be collected and sensitivity analyses conducted: (i) self-referral or referral from a non-general practice source recorded in advocacy service logs, (ii) clinician referral recorded in the advocate educators' records, (iii) referral to other domestic violence agencies. After the primary analysis based on referrals in the general practice record, we will conduct sensitivity analyses, combining these referrals sequentially with referrals in (i), (ii), (iii) and finally with all referral sources combined.

Cost data for the intervention are directly recorded: salaries and management costs of advocate educators, materials (posters and cards), practice payments for training sessions and travel costs. Data on domestic violence costs are based on secondary sources [5].

The 'Physician Readiness to Manage Partner Violence Survey' (PREMIS) questionnaire is completed by GPs and practice nurses in intervention and control practices at baseline, and again concurrent with outcome data collection one year later.

### Masking (blinding)

Due to the nature of the intervention, patients and practice staff cannot be blinded to allocation status, although patients are given no explicit information about the intervention. Research associates collecting outcome data could not be blinded to allocation status, but we have used a detailed flowchart for extraction to minimize this, plus referral to a panel for arbitration over difficult cases. We are also validating a sample of our data collection using a blinded researcher. All analyses will be conducted blind to treatment allocation.

### Sample size

With 24 intervention and 24 control practices, assuming a disclosure rate of 1% in control practices (a conservative estimate based on our survey of 12 Hackney practices [54] and an intra-cluster correlation coefficient of 0.03, we will be able to detect an increase of 5.2% in the disclosure rate with a power of 80% at a significance level of 0.05. This calculation assumes that there are on average 1600 women in the relevant age group in each practice, and also takes account of variation in cluster size. With this number of practices we will also be able to reliably detect a three-fold difference between intervention and control practices in the referral of women disclosing abuse to domestic violence advocacy services.

### Analysis

Analyses of referral and identification will be by intention to treat with a Poisson regression model. Our independent variable is the number of referrals/identifications for each cluster. The number of women aged 16 or older will be included as the exposure and practice will be included as a random effect. The analysis will be adjusted for minimization factors. Additionally the analysis will be adjusted for the practice baseline rate if considered to improve the precision of our treatment estimate and not adversely affecting the fit of the model.

Directly collected cost data for the intervention will be combined with service costs and costs of domestic violence in relation to imputed benefits of advocacy referral in a Markov model to calculate cost effectiveness in terms of cost per quality adjusted life years. We have used this approach in a pilot study for this trial [55].

Before-and-after analyses of scores for each of the three sections of the PREMIS questionnaire (knowledge, opinion, practice) will be carried out using descriptive statistics and multivariable models (including respondent characteristics) to test for the effect of the intervention.

## Discussion

This is the first European randomised controlled trial of an intervention to improve the health care response to domestic violence. The intervention is a collaboration between primary health care services and third sector agencies specialising in domestic violence, combining educational outreach training sessions in general practices with a referral pathway to a named advocate-educator who also was part of the team delivering the training. Additional components of the intervention included a prompt to ask about domestic violence embedded in the medical record and continuing audit and feedback on disclosure and referral data. The primary outcome measure- referral to domestic violence advocacy - is an intermediate outcome, on a causal pathway towards reduced violence, and improved quality of life and mental health for women who are referred. We will put our results into a model that extrapolates to those outcomes, which will estimate the cost-effectiveness of the intervention. As health care services in the UK and internationally are moving to address domestic violence [56], particularly through training of clinicians, our findings - based on a pragmatic trial - will have the potential to influence health service policy. If they are positive, then the IRIS model could be implemented within primary care. If they are negative, then other interventions to improve identification and referral of women experiencing domestic violence need to be developed.

## Ethics Approval

The study has ethical approval from the South East Research Ethics Committee (REC Reference: 07/MRE01/65).

## Competing interests

Annie Howell and Medina Johnson are domestic violence advocate educators. Positive trial findings would support their career development. The other authors have no competing interests.

## Authors' contributions

GF and RAD had the original idea for the study. GF, RAD, DS, JR, CR and SE designed the trial; MJ, AH, RAD and GF designed the intervention; MJ and AH deliver the intervention; DD and AG collect the data; CR analyses the data supervised by SE, AD performs the cost-effectiveness analysis, AG wrote the first draft of this paper and all authors contributed towards the revision and the refinement of the final manuscript. All authors read and approved the final manuscript. GF is the guarantor of the paper.

## Pre-publication history

The pre-publication history for this paper can be accessed here:

http://www.biomedcentral.com/1471-2458/10/54/prepub
